# Racism-related stress and mental health among black women living in Los Angeles County, California: A comparison of postpartum mood and anxiety disorder screening scales

**DOI:** 10.1007/s00737-024-01458-w

**Published:** 2024-04-02

**Authors:** Kortney Floyd James, Keren Chen, Sasha S. Hindra, Sydney Gray, Milllicent N. Robinson, Courtney S. Thomas Tobin, Kristen Choi, Denise Saint Arnault

**Affiliations:** 1 School of Nursing, University of California Los Angeles, Los Angeles, CA USA; 2 RAND Corporation, Santa Monica, CA USA; 3 David Geffen School of Medicine, Department of Medicine Statistics Core, University of California, Los Angeles, Los Angeles, CA USA; 4 University of California Irvine, Sue & Bill Gross School of Nursing, Irvine, CA USA; 5 Long Beach, CA USA; 6 School of Social Work, University of North Carolina at Chapel Hill, Chapel Hill, NC USA; 7 Jonathan and Karin Fielding School of Public Health, Department of Community Health Sciences), University of California Los Angeles, Los Angeles, CA USA; 8 Fielding School of Public Health, Department of Health Policy and Management, University of California Los Angeles, Los Angeles, CA USA; 9 School of Nursing, Department of Health Behavior and Biological Sciences, University of Michigan, Ann Arbor, MI USA

**Keywords:** Black women, Mental health, Racism, Postpartum, Motherhood

## Abstract

**Purpose:**

To assess Black women’s exposure to and appraisal of racism-related stress during the postpartum period and to distinguish its impact on three indicators of postpartum mood and anxiety disorders (PMADs) symptoms.

**Methods:**

Data from the Black Mothers’ Mental Wellness Study (N = 231) and linear regression models estimated the associations between racism-related stress and the PMAD indicators: 3-item Edinburgh Postnatal Depression Scale (EPDS-3), 8-item Patient Health Questionnaire (PHQ-8), and PHQ-15.

**Results:**

The majority of participants (80.5%, N = 186) experienced racism a few times a year or more, of which 37.1% (N = 69) were bothered somewhat and 19.3% (N = 36) a lot. Racism-related stress, income, level of education, and history of mental health diagnosis explained greater variance in PMAD symptoms as measured by the PHQ-8 score (R^2^ = 0.58, *p* =  < 0.001) compared to the EPDS-3 (R^2^ = 0.46, *p* =  < 0.001) or the PHQ-15 (R^2^ = 0.14, *p* = 0.035).

**Conclusions:**

Racism is a stressor for Black women living in Los Angeles County, California. Racism-related stress and emotional expression of PMAD symptoms were salient to the postpartum mental health of the Black women in this study. Findings from this study suggest that the PHQ-8 should be used to assess how racism impacts Black women’s postpartum mental health.

Known factors increase all women’s risk of developing perinatal mood and anxiety disorders (PMADs) (e.g., prenatal depression, lack of social support, stressful life events) (Ko et al. 2012). However, it is unclear whether racism influences Black women’s PMAD risk. While the Southern United States (US) is historically associated with anti-Black racism, Los Angeles County, California (LAC) also has a significant history of racism. Racial discrimination in LAC dates back to 1910 when Black people migrated westward from the South (Angeles and Anti-Racism 2023). Policies and police enforcement perpetuated segregation despite the Civil Rights Act of 1964, with communities like Compton and Culver City enforcing sundown town practices, which are all-White neighborhoods that ensured non-White people did not live or stay after dark by legislative means or violence (Angeles and Anti-Racism 2023). Structural and interpersonal racism in LAC affects Black women impacting their interaction with healthcare systems (Cummings 2022). Despite substantial evidence of racism’s role as a stressor leading to adverse mental health outcomes among Black women (Burke et al. 2023; Hill and Hoggard 2018; Liao et al. 2020; Scott et al. 2023), studies often overlook it as a factor affecting postpartum mental health.

LAC, the most populous county in the US, exhibits stark disparities, with Black women comprising 9% of the population but representing 38% of those experiencing PMAD symptoms (Health 2017). Suppression of emotional PMAD symptoms, often due to cultural expectations such as the Strong Black Woman/Superwoman role (Woods-Giscombé 2010), can lead to somatic symptoms (Evagorou et al. 2016). Racism-related stress (RRS), the psycho-emotional injury caused by experiencing racism (Carter 2006), can also contribute to PMAD symptoms among Black women. While the negative effects of RRS on the mental health of Black adults (Sellers et al. 1998; Carter 2006), including the prenatal period (Ertel et al. 2012) are well-established, its specific impact on postpartum mental health remains unclear.

In this study, we integrated the *Conceptual Model* of *how Racism Operates and Results in Inequities in Maternal Morbidity and Severe Maternal Mortality* (CMOR) (Hardeman et al. 2022) and Social Stress Theory (SST) (Pearlin 1999; Pearlin et al. 1981) to assess the relationship between RRS and postpartum mental health among Black women. Hardeman et al. (2022) underscored the complex mechanisms through which racism disproportionately impacts the perinatal outcomes of Black women. SST highlights how individuals’ social status influences their exposure to social stressors, making Black women more susceptible to RRS. Given the high prevalence of PMADs among Black women in LAC and the pervasive nature of racism, this study aimed to evaluate Black women’s experiences with RRS and its impact on three indicators of PMAD symptoms. We addressed two main objectives: (1) Assess Black women’s exposure to and appraisal of RRS during the postpartum period; (2) Examine the associations between RRS and three indicators of PMAD symptoms: the 3-item Edinburgh Postnatal Depression Scale (EPDS-3), 8-item Patient Health Questionnaire (PHQ-8), and 15-item PHQ.

## Methods

### Design

**Fig. 1:**
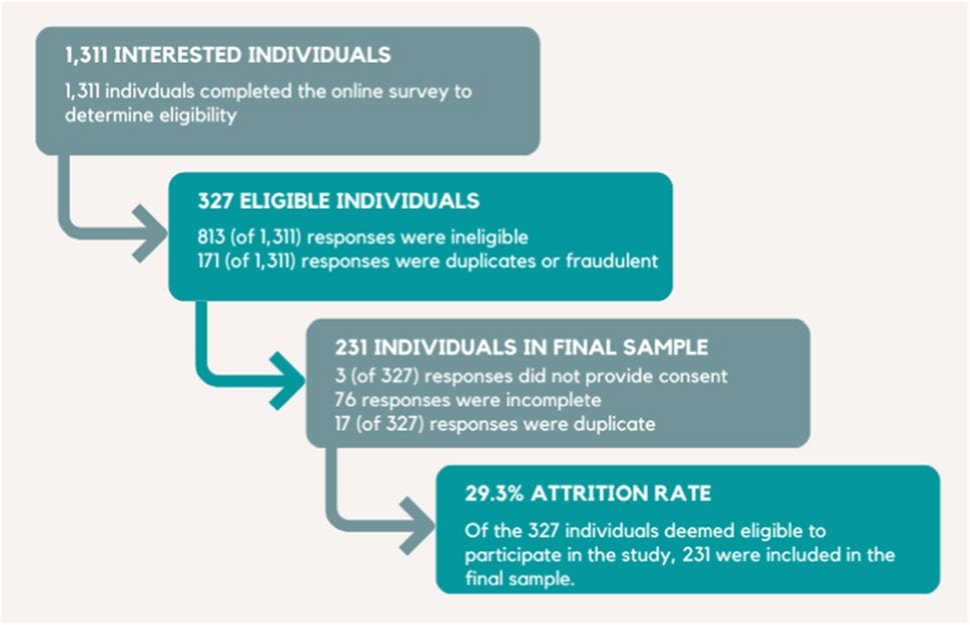
Sample flow diagram

The Black Mothers’ Mental Wellness Study used a sequential explanatory mixed methods design to investigate and describe the relationships between RRS and PMAD symptoms among Black women living in LAC; this analysis reports quantitative findings. Data were collected between August 2022, and April 2023. Eligibility criteria included women who: (a) self-identified as Black, (b) were ≤ 18 years, (c) lived in LAC, (d) were 6 weeks -12 months postpartum, and (e) had a singleton birth. Approval from University of California, Los Angeles IRB was obtained. Recruitment occurred throughout LAC using physical and digital flyers posted via websites, social media, and within establishments in locations that served Black women (e.g., mental health clinics, pediatric offices, community organizations). Potential participants used a URL to complete eligibility screening questions. Eligible participants received a unique URL via email to the consent form and online Qualtrics survey. Participants received a $15 VISA gift card for completing the survey. Based on linear multiple regression analysis with a power of 0.80, 0.06 effect size (f2), five predictor variables, at an alpha level of 0.05, the recommended sample size was at least 220 participants; the final sample included 231 complete survey responses (Fig. 1).

## Measures

### Symptoms of PMADs

As Black women may experience emotional and somatic PMAD symptoms (Walton and Shepard Payne 2016), only assessing one dimension may lead to inaccurate or underdiagnosis (Payne 2012). Therefore, three PMAD symptom screening tools were used Table 1.

**Table 1:** Description of scales to measure symptoms of perinatal mood and anxiety disorders

Concept	Scale	Example of items and response options	Total score range	Interpretation
Postpartum anxiety and depressive symptoms	3-item Edinburgh Postnatal Depression scale	“I have blamed myself unnecessarily when things went wrong.” “I have been anxious or worried for no good reason.” “I have felt scared or panicky for no very good reason.” Response choices ranged from “0 No never or not at all” to “3 Yes, very often, quite a lot, or most of the time	0 – 9	A total score of 3 – 5 may indicate the presence of depression. A total score of 6 or higher indicates a higher probability of depression
	8-item Patient Health Questionnaire	“How often have you been bothered by: Feeling bad about yourself – or that you are a failure or have let yourself or your family down? Trouble concentrating on things, such as reading the newspaper or watching television? Moving or speaking so slowly that other people could not have noticed? Or the opposite – being fidgety or restless that you have been moving around a lot more than usual?” Response choices ranged from: 0 to 1 day “not at all,” 2 to 6 days “several days,” 7 to 11 days “more than half the days,” and 12 to 14 days “nearly every day.”	0 – 24	A total score of 0 – 4 indicates no depressive symptoms, 5 – 9 mild depressive symptoms, 10 – 14 moderate depressive symptoms, 15 – 19 moderately severe depressive symptoms, 20 – 24 severe depressive symptoms
Somatization of postpartum anxiety and depressive symptoms	15-item Patient Health Questionnaire	“How much have you been bothered by any of the following problems? Stomach pain, back pain, pain in your arms, legs, or joints (knees, hips, etc.), chest pain, dizziness, trouble sleeping?” Response choices ranged from “0 not bothered at all, 1 bothered a little, 2 bothered a lot.”	0 – 30	A total score 0 – 4 represents minimal somatization, 5 – 9 low somatization, 10 – 14 medium somatization, 15 – 30 high somatization

#### 3-item Edinburgh Postnatal Depression Scale

The EPDS-3 (α = 0.85) measured symptoms of anxiety and depression in the past seven days (Kabir et al. 2008; Smith-Nielsen et al. 2021). The EPDS-3 is more sensitive (95%) and tends to identify more women (specificity 98%) experiencing anxiety and depression than the 10-item version (Kabir et al. 2008). The EPDS-3 has been validated in Black American women 1 week to 9 months postpartum (Lee King 2012).

#### 8-item Patient Health Questionnaire

The PHQ-8 (α = 0.87) was used to assess the severity of participants’ depressive symptoms over the past two weeks. The PHQ-8 omits suicidal ideation; because this survey was administered online researchers could not quickly intervene, if necessary. The PHQ-8 was included due to previous research suggesting the EPDS may be inefficient in identifying Black women who may be experiencing PMADs (Floyd James et al. 2021). In clinical practice, the PHQ-8 is a reliable tool (sensitivity and specificity 88%) (Kroenke et al. 2009), and has been validated in Black postpartum mothers (Pavlov et al. 2022).

#### 15-item Patient Health Questionnaire

Because previous literature suggests Black women may experience physical symptoms of depression or anxiety, the PHQ-15 was included to measure participants’ somatization of PMADs over the past 4 weeks (Kocalevent et al. 2013). The PHQ-15 has been used in clinical practice to detect somatization in those experiencing psychological distress. Clinically, the reliability of the PHQ-15 is questionable because many symptoms are correlated with physical conditions. However, in this study the PHQ-15 showed high reliability (α = 0.79), which is like previous research that included Black postpartum women (α = 0.73) (Wilkie et al. 2018).

### Racism-related stress

The Racism and Life Experience Scale—Daily Life Experiences subscale (RaLES-DLE) was developed in 1997 to assess the frequency, perception, and stressfulness of racism and its impact on biopsychosocial outcomes (Baron et al. 2007; Harrell et al. 1997). The RaLES-DLE assesses not only the frequency of interpersonal racism over the past year, but also the self-appraisal of those acts, which may explain differing perceptions of racism and PMAD symptoms within the same group (i.e., Black women) (Brown et al. 2020, 2018; Clark et al. 1999; Pearlin et al. 1981). The 20-item scale (α = 0.95) has 2-part questions that capture (1) how often one feels discriminated against due to their race in their daily lives (i.e., “Being mistaken for someone who serves others, such as a janitor…”, “Being insulted, called a name, or harassed”) with response options ranging from “0 – never” to “5—once a week or more”; and (2) one’s self-appraisal of the racist encounter (i.e., “How much does it bother you?”) with response options ranging from “0—has never happened to me” to “5—bothers me extremely.” Higher scores indicate higher frequency and levels of distress.

### Sociodemographic characteristics

Sociodemographic characteristics were included as covariates, given their documented influence on perinatal mental health (Ghaedrahmati et al. 2017; Silverman et al. 2017): *Age* (continuous), *number of dependent children* (continuous), *death of any children* (no, yes), *birth type* (cesarean, vaginal), *history of miscarriage* (no, yes), *educational level* (did not complete high school, General Education Diploma (GED), high school diploma, Associate degree, Bachelor’s degree, Master’s degree, Doctorate degree), *monthly income* (less than $1,000; $1,000–$1,999; $2,000-$2,999; $3,000–$3,999; $4,000 to $4,999; $5,000 or more), *relationship status* (single/never married, married/living with a partner, divorced/separated, widowed), and *history of any mental health diagnosis prior to recent pregnancy* (i.e., anxiety, bipolar disorder, depression, postpartum depression, schizophrenia) (no, yes).

## Data analysis

To assess Black women’s exposure to and appraisal of RRS, frequency scores of racism experiences were averaged from the original 20-item RALES-DLE frequency scale. Scores were then recoded into a three-category frequency variable (“less than once a year”, “a few times a year”, “at least once a month”). This recoding was based on thresholds 1 and 3, as defined by the original frequency scale, where 1 corresponds to “a few times a year” and 3 corresponds to “about once a month.” The stress scores were averaged from the original 20-item RALES-DLE self-appraisal stress scales and were recoded into the three-category variable (“a little”, “somewhat”, “a lot”) based on the threshold 1.5 and 2.5, as defined by the original stress scale, where 1 corresponds to “bothers me a little”, 2 corresponds to “bothers me somewhat” and 3 corresponds to “bothers me a lot.” The thresholds were selected according to the original design of the response options. The final RaLES-DLE score that was used in the regression models was calculated by summing the products of the frequency scale and stress scale for all 20 items and normalizing it (mean = 0, SD = 1).

Simple linear regression analyses were used to examine relationships between RRS (final RaLES-DLE score) and PMAD symptoms, as measured by three scales, EPDS-3, PHQ-15, and PHQ-8, respectively. Then multivariable models were adjusted by age, education, income, mental health diagnosis, birth type, miscarriage experience, death of children, number of children, and relationship status. Candidate variables included in the models were selected based on known and hypothesized association from literature. The ordinal variables for education and income were treated as numeric variables to test for the linear trend of the effect. We compared adjusted R-squared values to assess how well the RaLES-DLE score explained variance in each of the three PMAD outcome measures.

To assess the influence of RRS on the development of PMADs, a sensitivity analysis was performed. This involved dichotomizing the scores from PMAD scales at their clinical cutoffs for moderate risk of depression: a score of 3 or higher on the EPDS-3, and 10 or higher on both the PHQ-8 and PHQ-15. Multivariable logistic regression models were then employed to compare the RaLES-DLE scores across the two groups defined by these cutoffs. The predictive accuracy of these models was evaluated using the area under the receiver operating characteristic (ROC) curve (AUC), providing a measure of their performance in distinguishing between the groups. Two-sided p-values associated with the regression coefficient less than 0.05 were considered statistically significant. All analyses were performed using R Statistical Software (v4.2.3; R Core Team 2023).

## Results

On average, participants were 31 years old (SD = 4.2), 7.1 months postpartum (SD = 4.2), and had two children. Table 2. Most of the sample had cesarean births (N = 178, 77.1%), was married/living with their partner (N = 205, 88.7%) and had a college degree (N = 185, 80.1%). RaLES-DLE scores indicated that 80.5% (N = 186) of participants experienced racism a few times a year or more, of which 37.1% (N = 69) were bothered somewhat and 19.3% (N = 36) were bothered a lot (Table 3). Overall, there were low to mild symptoms of PMADs in the sample, as indicated by average EPDS-3 scores of 4.26 (possible anxiety and depression), average PHQ-8 scores of 6.68 (mild depressive symptoms), and average PHQ-15 scores of 7.51 (low somatization).

**Table 2:** Demographic and reproductive health characteristics of the sample (N = 231)

Sample Characteristic	N, % (Mean ± SD)
Age (year)	31.0 ± 4.2
Infant age (month)	7.1 ± 4.2
Number of children given birth to	1.7 ± 0.8
Any death of children	
No	206, 89.2%
Yes	25, 10.8%
Birth type	
Vaginal birth	53, 22.9%
Cesarean birth	178, 77.1%
Miscarriage	
No	180, 77.9%
Yes	51, 22.1%
Employment status	
On maternity leave	50, 21.7%
Unemployed	23, 10.0%
Self-employed	16, 6.9%
Part-time/occasional	69, 29.9%
Full-time	73, 31.6%
Insurance	
No	26, 11.1%
Yes	205, 88.9%
Education	
Did not complete high school	2, 0.9%
GED	3, 1.3%
High school diploma	41, 17.8%
Associate degree	68, 29.4%
Bachelor’s degree	99, 42.9%
Master’s degree	14, 6.1%
Doctorate Degree	4, 1.7%
Income	
Less than $1,000	21, 9.1%
$1,000 to $1,999	44, 19.1%
$2,000 to $2,999	51, 22.1%
$3,000 to $3,999	69, 29.9%
$4,000 to $4,999	24, 10.4%
$5,000 or more	21, 9.1%
Relationship status	
Single/Never married	13, 5.6%
Married/Living with a partner	205, 88.7%
Divorced/Separated	12, 5.2%
Widowed	1, 0.4%
Frequency of racism experience	
Less than once a year	45, 19.5%
A few times a year	152, 65.8%
At least once a month	34, 14.7%
Mental health history	
Positive mental health diagnosis	56, 24.2%
Negative mental health diagnosis	175, 75.8%
	Mean ± SD (min – max)
PMAD symptoms	
EPDS-3	4.3 ± 2.5 (0 – 9)
PHQ-8	6.7 ± 5.2 (0 – 24)
PHQ-15	7.5 ± 4.9 (0 – 22)

**Table 3:** Frequency of racism experiences and self-appraisal (N = 231)

	Self-appraisal (stress) of racist encounter		
Frequency of racist encounters	Bothered a little	Bothered somewhat	Bothered a lot
Less than once a year	45 (100.0%)	0 (0%)	0 (0%)
Few times a year	81 (53.3%)	65 (42.8%)	6 (4.0%)
At least once a month	0 (0%)	4 (5.8%)	30 (88.2%)

Unadjusted linear regression models showed that RaLES-DLE score had a significant positive association with all three PMAD scales. The adjusted R-squared for Model 1 to Model 3 were 0.46, 0.58, and 0.14, respectively. These results were robust in adjusted models that controlled for age, education, income, birth type, history of miscarriage, death of children, number of children, relationship status, and history of mental health condition. The multivariable regression analyses revealed that the RaLES-DLE score (*p* < *0.001),* education (*p* = *0.01*), and mental health diagnosis history (*p* < *0.001*) were positively associated with the EPDS-3 total score; income (*p* = *0.009*) was negatively associated (Table 4**, **Model 1). RaLES-DLE score (*p* < *0.001*), education (*p* = *0.007),* and mental health diagnosis history (*p* = *0.01*) were positively associated with the PHQ-8 total score, while income (*p* < *0.001)* was negatively associated (Table 4, Model 2). Lastly, one-standard deviation increase in RaLES-DLE score (*p* = *0.03)* was positively associated with a 0.01 increase in the PHQ-15 total score, while income (*p* = *0.004)* was negatively associated; being divorced/separated *(p* = *0.03)* had an average of 3.1 unit increase in PHQ-15 total score compared to those that are married or living with a partner (Table 4, Model 3).

**Table 4:** Multivariable regression models to assess factors associated with PMAD symptoms, as measured by three scales (N = 231)

^1^Outcome	Variable	b	SE	95% CI		*p*-value
				LL	UP	
^2^Model 1: EPDS-3	^3^RaLES-DLE scores, per 1 SD	1.364	0.152	1.064	1.664	< 0.001***
	Age, per 1 year	-0.002	0.036	-0.072	0.068	0.965
	Education, per category	0.390	0.157	0.083	0.698	0.013*
	Income, per category	-0.298	0.113	-0.520	-0.076	0.009**
	Mental health diagnosis history (Yes)	1.243	0.330	0.595	1.890	< 0.001***
	Birth type (C-section)	-0.468	0.311	-1.077	0.142	0.134
	Miscarriage (Yes)	-0.511	0.339	-1.176	0.154	0.133
	Death of children (Yes)	0.445	0.435	-0.408	1.298	0.307
	Number of children, per child	0.039	0.188	-0.329	0.407	0.835
	Relationship (Single/Never married)	-0.279	0.608	-1.469	0.912	0.646
	Relationship (Divorced/Separated)	-0.778	0.596	-1.946	0.390	0.193
	Relationship (Widowed)	0.288	2.059	-3.748	4.325	0.888
^2^Model 2: PHQ-8	^3^RaLES-DLE scores, per 1 SD	3.265	0.270	2.732	3.797	< 0.001***
	Age, per 1 year	0.033	0.063	-0.091	0.157	0.602
	Education, per category	0.758	0.279	0.212	1.304	0.007**
	Income, per category	-0.752	0.201	-1.147	-0.358	< 0.001***
	Mental health diagnosis history (Yes)	1.417	0.586	0.268	2.566	0.016*
	Birth type (C-section)	0.571	0.552	-0.510	1.652	0.301
	Miscarriage (Yes)	-0.0003	0.602	-1.181	1.180	0.999
	Death of children (Yes)	0.843	0.772	-0.671	2.356	0.276
	Number of children, per child	-0.580	0.333	-1.233	0.073	0.083
	Relationship (Single/Never married)	-1.711	-3.824	-3.824	0.402	0.114
	Relationship (Divorced/Separated)	-0.071	-2.144	-2.144	2.001	0.946
	Relationship (Widowed)	1.860	-5.303	-5.303	9.023	0.611
^2^Model 3: PHQ-15	^3^RaLES-DLE scores, per 1 SD	0.779	0.368	0.054	1.504	0.035*
	Age, per 1 year	0.118	0.086	-0.052	0.288	0.171
	Education, per category	-0.099	0.379	-0.847	0.648	0.793
	Income, per category	-0.790	0.274	-1.330	-0.251	0.004**
	Mental health diagnosis history (Yes)	0.394	0.798	-1.179	1.967	0.621
	Birth type (C-section)	0.984	0.751	0.496	2.464	0.191
	Miscarriage (Yes)	0.073	0.820	-1.543	1.689	0.928
	Death of children (Yes)	-0.925	1.051	-2.997	1.147	0.379
	Number of children, per child	-0.405	0.453	-1.299	0.488	0.372
	Relationship (Single/Never married)	0.927	1.467	-1.966	3.819	0.528
	Relationship (Divorced/Separated)	3.059	1.439	0.223	5.895	0.034*
	Relationship (Widowed)	-0.683	4.974	-10.487	9.121	0.890

95% CI = 95% Confidence Interval

^1^Degree of Freedom = 212; dependent variables were the sum of scores of EPDS-3, PHQ-8, and PHQ-15

^2^Adjusted R-squared were 0.46, 0.58, and 0.14, respectively

^3^Normalized sum of RaLES-DLE scores

In the sensitivity analysis, multivariable logistic regression models revealed that an increase of one standard deviation in RRS scores was associated with a substantial increase in the likelihood of exhibiting a positive indicator for moderate depression. Specifically, this increase corresponded to a 3630% rise in the odds of scoring within the moderate depression range on the EPDS-3 score (p < 0.001, AUC = 0.835), and a 449% increase in the odds for the PHQ-8 score (p < 0.001, AUC = 0.867). However, the analysis did not demonstrate a significant association between RRS and the moderate depression indicator as measured by the PHQ-15 score (p = 0.661, AUC = 0.617), suggesting that the impact of RRS may vary depending on the depression assessment tool utilized.

## Discussion

We assessed Black women’s exposure to and appraisal of RRS during the postpartum period and its correlation with three PMAD symptom indicators: EPDS-3, PHQ-8, and PHQ-15. The findings highlight the relevance of CMOR and SST in illustrating how social position influences RRS exposure among Black women.

### Black women’s exposure to and appraisal of RRS

Most participants (N = 152, 65.8%) reported experiencing interpersonal racism a few times a year, of which 46.8% were bothered somewhat to a lot. These findings underscore the insidious nature of RRS among Black women A previous study (Chambers et al. 2020) found that almost half of the Black pregnant and postpartum women reported experiencing and being affected by racism, a higher proportion than the current study. Additionally, Black women with higher incomes in racially diverse and wealthy zip codes in Oakland, California experienced less racial discrimination than those in economically deprived areas. Despite not measuring neighborhood characteristics this sample’s high education and income levels may have influenced RRS levels, aligning with SST, which suggests marginalized individuals face greater exposure to social stressors (Robinson et al. 2023). This study captured experiences of interpersonal racism in public settings, unlike previous research (Chambers et al. 2020) which captured various levels of racism, possibly explaining the lower RRS here. Future research should examine different levels of racism and RRS in a more diverse sample to determine their impact on PMAD symptoms.

### RRS and PMAD symptoms

Findings revealed that when controlling for sociodemographic variables that influence perinatal mental health, increased exposure to RRS was generally associated with higher PMAD symptoms among Black women. These findings reflect that, similar to previous research about RRS in Black women (non-postpartum) (Burke et al. 2023; Scott et al. 2023), RRS negatively impacts postpartum mental health. However, the clinical significance is minor and would not have much effect on one’s total PMAD score. These minor increases may be due to the overall low frequency and appraisal of RRS, as described above, and PMAD symptom scores. They could also be attributed to the multileveled manifestations through which racism ultimately shapes perinatal health outcomes among Black women. As highlighted in the CMOR, racism influences health via a host of structural, institutional, interpersonal, and internalized pathways (Hardeman et al. 2022), of which exposure to RRS is just one. Despite the relatively low exposure to RRS reported in the present study, the pernicious impact of racism in the lives of Black women is well-documented (Scott et al. 2023) and additional research is needed to pinpoint the specific mechanisms through which it contributes to heightened PMAD symptoms among this population.

### Comparison of RRS across multiple pmad screening tools

Multivariate models revealed that RRS, income, level of education, and positive history of a mental health diagnosis explained 46% and 58% of the variation in PMAD symptoms, as measured by the EPDS-3 and PHQ-8, respectively; there was no significant association between RRS and PMAD symptoms indicated by the PHQ-15. While previous research yielded similar results regarding the association between PMAD symptoms and RRS (Bower et al. 2023; Segre et al. 2021), this study is the first to evaluate commonly used PMAD screening tools. The findings of this study reflect that these factors (RRS, income, level of education, and mental health history) are salient to the mental health of postpartum Black women and may be ideal to include in a holistic, culturally relevant PMAD screening tool and other interventions to support the mental health of Black postpartum women; however, further research is warranted. Conversely, these factors only accounted for 14% of the variance in PMAD symptoms as measured by the PHQ-15, which suggests that the women in this study experienced emotional symptoms of PMADs, as captured by EPDS-3 and PHQ-8. Previous research that found high levels of somatization in Black participants utilized Beck’s Depressive Inventory (Beck et al. 1988), did not include postpartum women (Ayalon and Young 2003), and restricted their sample to participants experiencing major depression, as determined by the Diagnostic and Statistical Manual of Mental Disorders (Brown et al. 1996). It is possible that the participants in the present study did not have high levels of somatization due to their overall low to mild PMAD symptoms, and Black women may experience postpartum anxiety and/or depression differently than at other points in their life. Overall, while findings suggest that the EPDS-3 and PHQ-8 should be used to assess how racism impacts Black women’s perinatal mental health, this study should be replicated in a larger sample of Black women with varying PMAD symptoms and throughout the perinatal period to determine the appropriate screening tools to assess RRS, PMAD severity, and somatization.

### Limitations

The study faced limitations in recruitment, design, and setting. Diverse strategies, including partnerships with community-based entities (Williams and Anderson 2021) were employed throughout LAC to facilitate successful recruitment. However, unacknowledged institutional harms from California’s academic medical institutions (Ralph J. Bunche Center Community Engagement Project Research Team 2022) may have affected potential participants’ trust in the research team’s intent (Le et al. 2022). The cross-sectional design precludes causal inference, and the findings’ generalizability is restricted to LAC residents. The exclusive focus on interpersonal racism may underestimate the broader racism experienced by Black women. The events surrounding the unjust murder of George Floyd in 2020 may have affected participants’ perceptions of racism. Validated PMAD measures were used, which is a study strength, but some instruments lack the ability to distinguish between anxiety and depression. Future studies may benefit from disaggregating these symptoms. Lastly, varying timeframes in PMAD measures necessitate caution in clinical interpretation, especially those with shorter assessment windows. While the findings in this study were likely not affected by these differences since separate analyses were conducted for each, clinicians should repeat screening, conduct a detailed health history assessment, and follow-up (O’Connor et al. 2016).

## Conclusion

This study advances our understanding of the harm of RRS in the lives of Black women and its role in perinatal mental health. Exposure to RRS was common in this sample of Black women and we found a robust association of RRS to PMAD symptoms, when measured using the EPDS-3 and PHQ-8, suggesting that these tools should be used to assess how RRS impacts Black women’s postpartum mental health. By developing comprehensive interventions that promote screening and identification of PMADs during the perinatal period with consideration for the unique experiences of Black women, there is potential to achieve health equity in perinatal mental well-being.

## Data Availability

Data is available upon request.
